# Psychometric validation of the informed consent assessment scale using item response theory and factor analysis

**DOI:** 10.3389/fmed.2025.1685730

**Published:** 2026-01-12

**Authors:** Nuno Silva Gonçalves, Pedro Morgado, Carlos Fernando Collares, José Miguel Pêgo

**Affiliations:** 1 Life and Health Sciences Research Institute (ICVS), University of Minho, Braga, Portugal; 2 ICVS/3B’s, PT Government Associate Laboratory, Braga, Portugal; 3 Clinical Academic Center, Braga, Portugal; 4 Medical Education Unit, Faculty of Medicine and Biomedical Sciences, University of Algarve, Faro, Portugal; 5 European Board of Medical Assessors, Cardiff, United Kingdom; 6 Inspirali Educação, São Paulo, Brazil; 7 Faculdades Pequeno Príncipe, Curitiba, Brazil

**Keywords:** assessment, communication, informed consent, item response theory, psycometric test, surgery

## Abstract

**Objectives:**

Informed consent is a central ethical and legal practice in medicine, yet communication skills specific to this task are under-assessed in undergraduate medical education. This study aimed to develop and validate the informed consent assessment scale (ICAS), a tool designed to evaluate communication competencies essential for delivering informed consent.

**Methods:**

This psychometric validation study was conducted over three academic years (2021–2023) and included 456 fifth-year medical students who completed a 10-min OSCE station on obtaining consent for right colectomy. The ICAS was developed through expert consensus using a structured focus group and qualitative assessment of content validity. Response process evidence was collected by querying assessors about their decision-making during scoring. Internal structure was examined using the exploratory factor analysis (EFA) with tetrachoric correlations, as well as the item response theory (IRT; Rasch and 2-parameter logistic (2PL) models). Reliability was assessed using Cronbach’s alpha, McDonald’s omega, and IRT-derived conditional reliability. Concurrent validity was evaluated through correlations with faculty and standardized-patient communication scores.

**Results:**

Parallel analysis supported a one-factor solution. The scale demonstrated essential unidimensionality (UniCo = 0.903, ECV = 0.807, and MIREAL = 0.235) and good model fit (RMSEA = 0.032, CFI = 0.966, and WRMR = 0.039). Reliability was high (McDonald’s *ω* = 0.841 and Cronbach’s *α* = 0.837). Q3 analysis indicated no local item dependence (mean Q3 = −0.037 and SD = 0.100). Item discrimination parameters in the 2PL model varied across items, enabling differentiation of student performance. ICAS scores showed strong correlations with global examiner ratings and moderate correlations with broader communication scales, supporting concurrent validity.

**Conclusion and practice implications:**

The ICAS is a valid and reliable instrument for assessing communication skills specific to informed consent. Its application in objective structured clinical examinations (OSCEs) provides actionable feedback for learners and supports curriculum efforts to strengthen ethically competent clinical communication.

## Introduction

Effective communication is a foundation of medical practice, supporting the doctor–patient relationship and exerting a significant influence on clinical outcomes. Despite its crucial role, communication skills (CSs) often receive less attention than cognitive and technical competence in medical education, resulting in suboptimal performance. The importance of CSs for medical professionals is well established, serving as a cornerstone to medical practice, as reflected in frameworks such as the Accreditation Council for Graduate Medical Education (ACGME) milestones and the CanMEDS framework (1, 2). Clear and empathetic communication is as essential as clinical, technical, and cognitive skills, positively impacting patients’ healthcare outcomes, satisfaction with medical care, treatment adherence, and even earlier detection of postoperative complications (3–11). Communication is a multidimensional skill that encompasses the ability to properly collect information from a patient, provide counsel, build a trusting relationship, understand the patient’s perspective, propose adequate treatment, engage patients in shared decision-making, and achieve consensus (7, 8, 12–14).

One of the emblematic moments in clinical encounters for CS is obtaining informed consent (IC) from a patient. The IC is a legal and ethical obligation, in which patients grant permission for doctors to perform a procedure (9, 15). The doctor must be able to explain the benefits, possible risks, and existing alternatives. IC benefits both the patient and the doctor since poor communication during this process can lead to misunderstandings, dissatisfaction, and even litigation against clinicians (16, 17). Despite its recognized importance, the literature demonstrates that patients are unsatisfied with the way IC is currently obtained, reporting undervalued patient autonomy and withheld knowledge by doctors (5, 15). In addition, medical professionals are also unhappy with their level of ability, citing a lack of formal training. Previous surveys have shown that even though residency directors considered the importance of including IC training in residency curricula, the majority of programs do not provide structured instruction in this particular domain (5, 9, 18–20). Many medical residents feel unprepared and unaware of proper IC delivery techniques (21). Many students and trainees rely almost exclusively on informal observation of senior clinicians or trial-and-error learning, with limited opportunities for formal instruction and feedback on their communication techniques. The incorporation of communication training, either during residency or during medical school, has been demonstrated to be effective and with positive results on doctor–patient relationship and, consequently, on patient care (3, 7, 8, 22, 23). Recognizing the lack of training during residency, the *Association of American Medical Colleges* included “Obtaining Informed Consent” in their 13 core entrustable professional activities (EPAs) “that all medical students should be able to perform upon entering residency” (24, 25).

Medical schools have a responsibility to train students to perform IC, yet current curricula often lack formalized instruction and assessment in this area. CS are usually taught and assessed in most medical schools using standardized patients and objective structured clinical examinations (OSCE), with excellent results (7, 12, 26–29). However, training and assessment specific to obtaining IC remain uncommon. The challenge of teaching and assessing communication skills, especially for high-stakes interactions like obtaining IC, is further complicated by the lack of standardized, validated tools to measure proficiency in this area.

Several tools are available to assess communication skills in medical education, including the Calgary–Cambridge Observation Guide, the Communication Assessment Tool, the Social Skills Inventory, and the SPIKES protocol for breaking bad news (14, 30–32). These frameworks provide valuable guidance on structuring conversations and ensuring patient-centered communication. However, their focus is broad, encompassing general clinical communication rather than addressing the specific challenges of IC. Furthermore, many of these tools lack the granularity necessary to evaluate the specific communication skills required for effectively obtaining IC.

Through the use of the item response theory (IRT), the authors proposed adapting the Minho communication assessment scale to evaluate the ability of medical students in obtaining IC (33). IRT allows for a more nuanced understanding of both student performance and the specific items on the scale, offering insights into item difficulty and discrimination (34, 35). This level of analysis is particularly relevant when validating a scale for IC, as it ensures that the tool not only assesses communication proficiency but also distinguishes between varying levels of competence. A validated communication skills assessment scale, specifically designed for obtaining IC, would allow for more targeted and constructive feedback. By identifying specific areas of strength and weakness, the scale would enable meaningful guidance to students, thereby improving their competency in this critical area.

Ultimately, this scale will provide medical educators with a standardized method to assess and improve students’ IC communication skills, aiming for better preparedness for autonomous practice. By enhancing these critical skills, the scale has the potential to enhance patient care, reinforce the doctor–patient relationship, and support safer, more informed decision-making.

## Methods

### Development of the scale

In light of the potential benefit for medical schools, particularly Portuguese medical schools, to include a summative OSCE station to assess obtaining IC from a standardized patient (SP), this study aimed to develop a customized assessment tool for this purpose. A thorough analysis of the current literature revealed no existing scale that met the intended criteria: written in Portuguese, assessor-friendly, and capable of distinguishing between non-competent and competent students.

The development of the ICAS followed an expert consensus process using a structured focus group. The panel included eight medical professionals representing a range of clinical specialties: one general practitioner, one general surgeon, one pediatric surgeon, three psychiatrists, one obstetrician/gynecologist, and one internist. All experts had at least 5 years of clinical experience (median 8 years) and were actively involved in undergraduate teaching, particularly in medical communication and OSCE assessment.

Each proposed item was discussed and independently rated for relevance and clarity. Items achieving ≥75% agreement among participants were retained for the final version. Items not reaching consensus were revised and re-evaluated in a second round of voting until agreement was achieved. Content validity was assessed qualitatively. This process ensured that the final set of items reflected both clinical and educational expertise in informed consent communication. This method ensured that the scale was developed using input from a diverse range of clinical perspectives, enhancing its relevance and applicability in various medical contexts. The final scale included 14 dichotomous items (Yes/No) and one global rating item scored from 1 (poor performance) to 5 (excellent performance; Table 1).

**Table 1:** Informed consent assessment scale (ICAS).

Number	Topic	Assessment
1	Starts the interview with an open-ended question. (Why did you come here today?)	Yes/No
2	Explores what the patient knows about their situation. (Why were the tests requested? Do you know why this particular test was ordered?)	Yes/No
3	Shares information appropriately (one piece of information at a time, using understandable language).	Yes/No
4	Explores the presence of emotions before continuing the interview (“What are you thinking/feeling?” “I imagine you might be worried/surprised by the result” “Perhaps you were not expecting this result”).	Yes/No
5	Validates the patient’s emotions (“It’s normal to feel worried about this situation”).	Yes/No
6	Uses appropriate strategies to respond to the patient’s emotions: silence, open-ended questions, empathetic comments, factual information.	Yes/No
7	Explains that the recommended treatment is surgical intervention (as an example).	Yes/No
8	Explains the risks associated with the surgical procedure.	Yes/No
9	Requests the informed consent signature in a timely and correct manner.	Yes/No
10	Makes themselves available to see the patient if needed.	Yes/No
11	Gives the patient the opportunity to discuss any additional questions.	Yes/No
12	Does not provide inappropriate or definitive information regarding the procedure or the pathology.	Yes/No
13	Does not defer responsibility for patient questions to others.	Yes/No
14	Asks the patient if they would like someone to pick them up OR whom they will talk to about the situation OR if they came accompanied.	Yes/No
15	Global assessment.	1 to 5

### Testing and application of the scale

The scale was applied across three consecutive OSCE examinations in a surgical rotation of the 5th year (of a 6-year MD program), involving a total of 456 students. In each exam, students were required to obtain IC for a surgical procedure (right colectomy for a colon cancer) from SP during a 10-min OSCE station. Each student was assessed by a faculty member trained in OSCE assessment and IC, for communication skills (using Minho-CAS) and for the IC skill (using the proposed scale), and by the SP for communication skills (using Minho-CAS). Following conventional recommendations for psychometric validation studies, a conservative 10:1 participant-to-item ratio was adopted as a general guideline for adequate sample size in scale development. Although this rule of thumb remains widely used, the literature indicates that the scale represents only an approximate guideline and may not account for factors such as item quality, dimensionality, and response distribution. In the present study, the total sample (*n* = 456) largely exceeded this minimum, ensuring stable parameter estimation and sufficient statistical power for both factor and item-response analyses (36, 37).

### Validation framework

Validation followed international recommendations for educational and psychological measurement, integrating four sources of evidence:

1. Test content (expert review)
2. Response processes
3. Internal structure (EFA and IRT)
4. Relations with other variables (concurrent validity).

### Statistical analysis

All analyses were conducted on the full dataset (*n* = 456).

This study considered item-level analysis essential for obtaining a more accurate measurement of a student’s ability to perform IC. Accordingly, IRT was employed to analyze and validate the scale. Prior to conducting IRT analysis, it was necessary to confirm the assumption of unidimensionality, ensuring that the scale assessed a single competency without influence from other unrelated factors. To evaluate unidimensionality, an exploratory factor analysis (EFA) was conducted using the FACTOR software (version 12.02.01), applying a tetrachoric correlation matrix suitable for dichotomous items. This study followed the same principles as previously reported, integrating the steps of evidence validation outlined by Cook et al. to ensure the robustness of the analysis (38).

### Test content

After the final draft, the scale was further reviewed and approved by additional expert medical professionals with extensive experience in communication skills assessment before its use in the OSCE. The panel included two surgeons (1 general, 1 pediatric), 2 internists, and 2 psychiatrists, each with at least 5 years of clinical experience and a minimum of 5 years of involvement in undergraduate medical education. All reviewers had prior experience in teaching or assessing communication skills in OSCE or clinical simulation settings. The reviewers evaluated each item for clarity, relevance, and representativeness using a structured qualitative checklist. No quantitative summary scores were generated. Minor wording adjustments were made based on their feedback before the final version of the ICAS was implemented. This process helped confirm that the scale was aligned with the intended content and adequately covered all necessary aspects of IC.

Response processes: To guarantee alignment between the assessment process and the intended construct, assessors were later questioned about their thought processes. They were asked whether they believed they were specifically evaluating informed consent and if they felt they were inadvertently assessing other competencies during the process. As assessors indicated alignment with the intended construct and no unintended competencies were being evaluated, no further changes to item content or scoring were required.

Internal structure: Applying IRT, which focuses on items as the unit of analysis and estimates the ability (also referred to as latent trait—*θ—*that represents their underlying proficiency in obtaining informed consent) by calculating specific item parameters such as difficulty and discrimination. These parameters were also used for item reduction analysis. For this analysis, both the Rasch model and the 2-parameter logistic model (2-PL) were applied. The Rasch model, a one-parameter logistic model, assumes that the probability of answering an item correctly is determined by the individual’s ability and the item’s difficulty. The 2-PL model incorporates both the item’s discrimination and difficulty indexes to unfold the student’s ability, expressed as a value of theta (θ). Model comparisons were conducted using: Akaike information criterion (AIC), Bayesian information criterion (BIC), sample-adjusted BIC (SABIC), Hannan–Quinn criterion (HQ), Log-likelihood (logLik), and likelihood ratio chi-square test. The local item dependence was further evaluated using Q3 residual correlations. To evaluate the internal consistency and reliability of the scale items, Cronbach’s alpha and McDonald’s omega were calculated.

Year-to-year comparisons: To explore cohort differences, chi-squared tests were conducted for dichotomous items and ANOVA or Kruskal–Wallis tests for continuous outcomes. *Post-hoc* analyses were performed using Tukey’s HSD or Dunn’s test where appropriate.

Concurrent validity: To ensure the accuracy of the results, the authors correlated the informed consent assessment scores with those from the communication assessment scale (CAS). While these scales do not measure the same construct, a high correlation was expected since informed consent is a specific form of communication. This analysis provided additional evidence for the validity of the scale and helped strengthen the overall findings. Furthermore, a correlation analysis and Cohen’s d were calculated between the test scores from the three assessed years to further support the validity of the results.

For the analysis, this study used *R version 4.3.1* with the mirt package, JASP (JASP Team, 2023; Version 0.18.1) [Computer software], FACTOR software version 12.02.01, and *jamovi* (Version 2.3.28), retrieved from https://www.jamovi.org.

## Results

### Informed consent assessment scale

A total of 456 students completed the informed consent OSCE station across three academic years using the final version of the ICAS (Table 1). All assessments were carried out by 12 trained faculty evaluators. No missing data were identified for ICAS scoring.

### Evidence based on internal structure

#### Exploratory factor analysis

The Kaiser–Meyer–Olkin (KMO) measure of sampling adequacy was 0.696, and the Bartlett’s test of sphericity was significant (χ^2^ = 5163.5, df = 91, *p* < 0.0001), supporting factorability of the correlation matrix.

Parallel analysis and examination of eigenvalues supported a unidimensional solution, with the first eigenvalue (*λ*₁ = 4.75) substantially larger than the second (λ₂ = 1.61), accounting for 33.9% of the total variance.

The overall indices also supported essential unidimensionality: unidimensional congruence (UniCo) = 0.903, explained common variance (ECV) = 0.807, and mean item residual absolute loadings (MIREAL) = 0.235. These values are close to, though slightly below, the strict thresholds for essential unidimensionality (UniCo >0.95, ECV > 0.85, and MIREAL <0.30), suggesting that the scale can be used as essentially unidimensional for IRT modeling (39, 40).

Global fit indices indicated good model fit, where the root mean square error of approximation (RMSEA) = 0.032, the comparative fit index (CFI) = 0.966, the goodness-of-fit index (GFI) = 0.989, the non-normed fit index (NNFI) = 0.952, and the weighted root mean square residual (WRMR) = 0.039.

#### Local item dependence

The Q3 residual correlations averaged −0.037 (SD = 0.100), and no item pair exceeded 0.20. These results demonstrated an absence of local dependence, thereby supporting the suitability of IRT modeling. For independent verification, the complete Q3 matrix is presented in Supplementary Table S1.

### Item response theory analysis

#### Rasch analysis

Rasch difficulty estimates exhibited meaningful variation across items (Table 2). The easiest items were related to addressing patient emotions (item 4), while the most difficult were those involving the explanation of surgical treatment (item 7). All items demonstrated acceptable fit, with infit values ranging from 0.738 to 1.157 and outfit values from 0.042 to 1.096.

**Table 2:** Item difficulty estimates from the Rasch model and the 2-parameter logistic (2PL) model, alongside discrimination estimates from the 2PL model.

Item	Rasch model	2-PL model	
	Difficulty	Difficulty	Discrimination
1	4.61	9.45	4.31
2	3.16	3.06	1.10
3	2.79	3.11	1.61
4	0.09	0.124	1.71
5	0.409	0.419	1.27
6	1.58	2.31	2.51
7	6.21	30.2	11.1
8	3.24	2.83	0.65
9	3.24	3.09	1.04
10	0.74	0.68	0.932
11	2.55	2.28	0.701
12	2.88	2.91	1.26
13	3.50	3.81	1.55
14	0.289	0.253	0.757

Comparison of item-level parameters estimated by both models.

**Figure 1:**
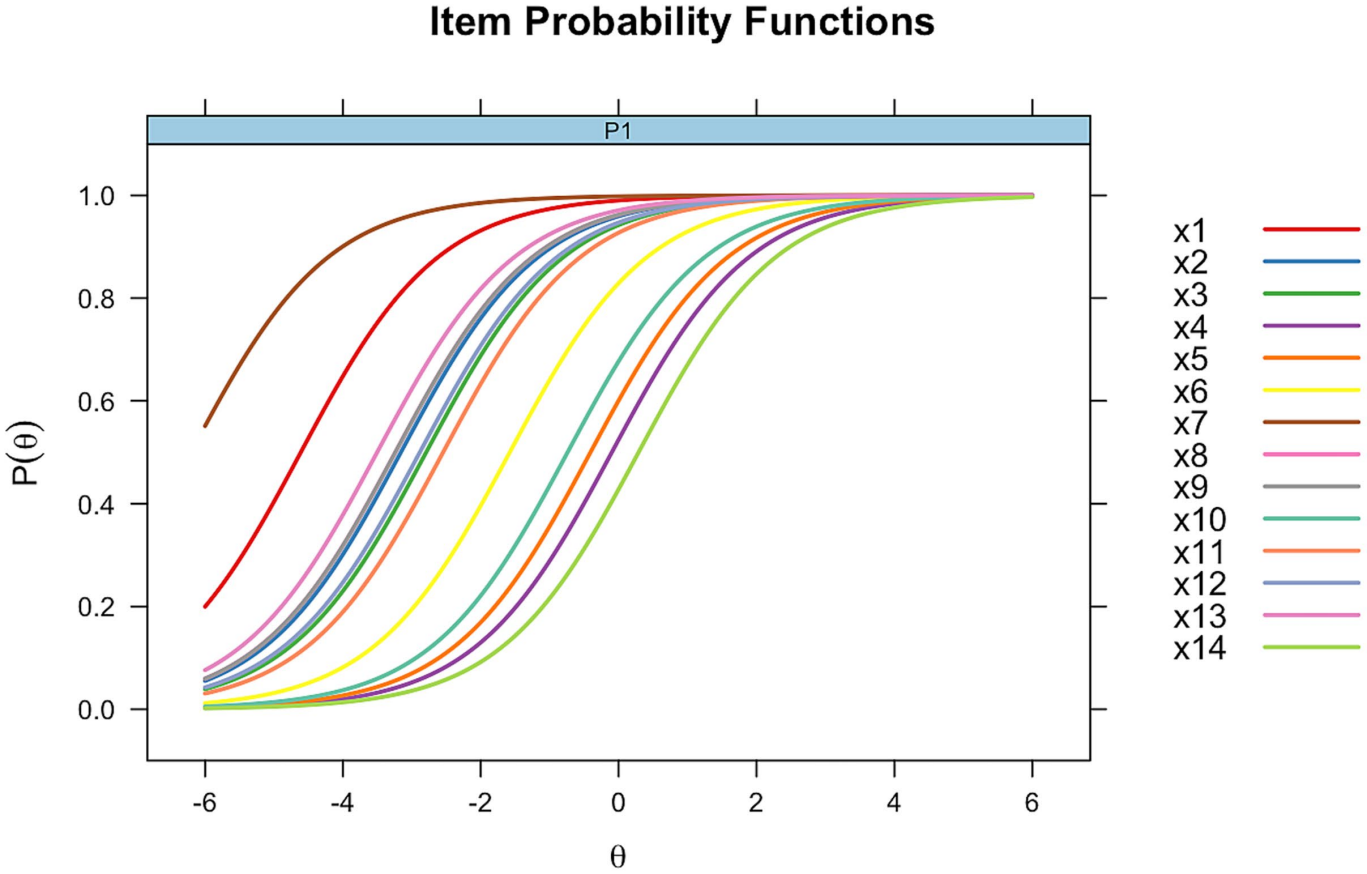
Item characteristics curve for Rasch analysis. Each curve represents one of the 14 items of the ICAS. Parallel curves indicate constant discrimination across items.

In Figure 1, the item characteristic curves (ICCs) for the Rasch model show parallel curves for all items, demonstrating the expected constant discrimination across items. The Rasch model estimates only difficulty for each item, with values ranging from 0.09 (item 4) to 6.21 (item 7). The model assumes that each item equally differentiates between ability levels, regardless of individual item characteristics.

**Figure 2:**
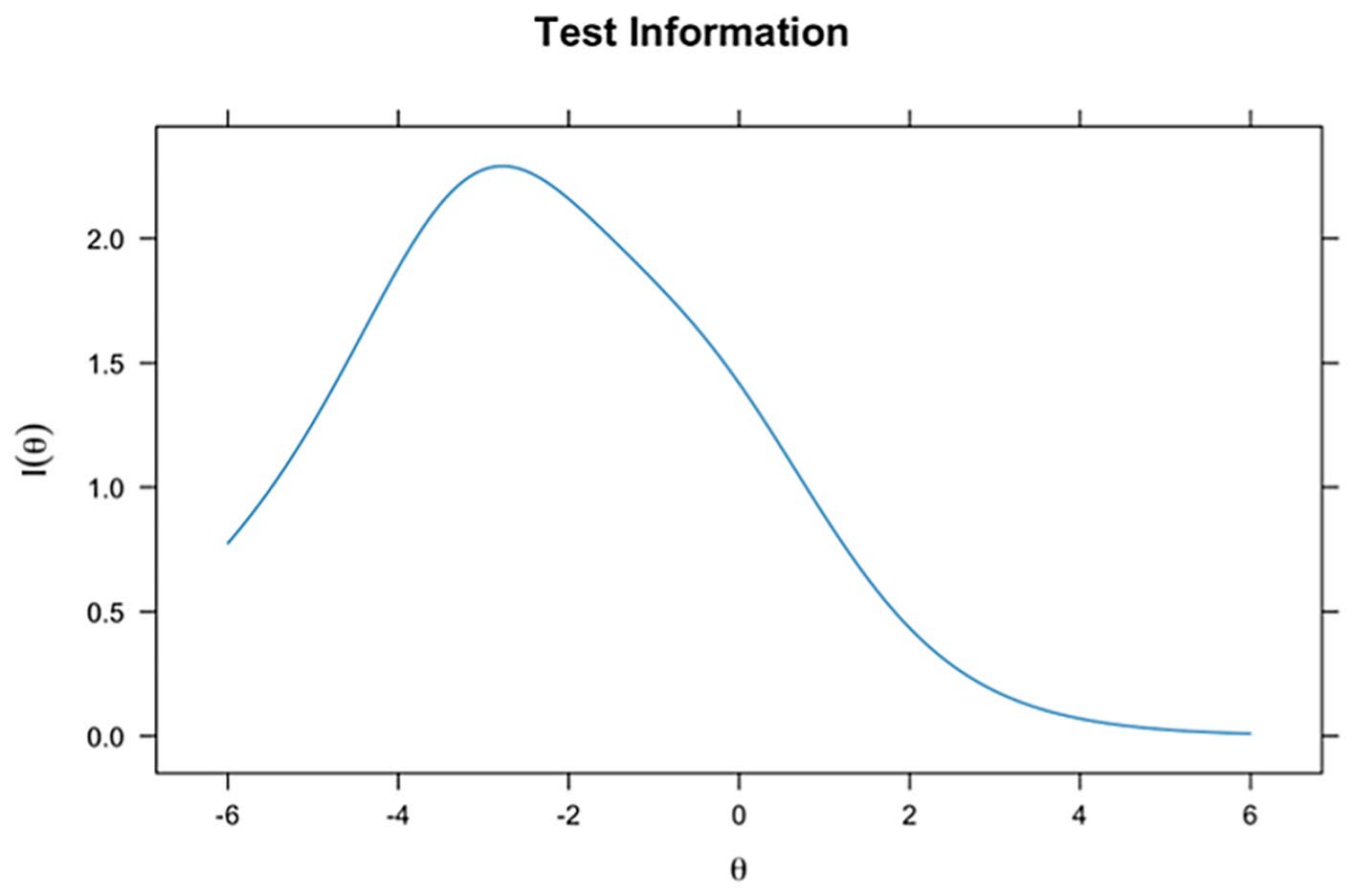
Item information curve for the Rasch model. The Rasch TIF peaks around *θ* = −2, indicating that the ICAS provides greater measurement precision for students with lower ability levels, where differentiating basic from inadequate performance is most critical.

The item information curve (TIF) for the Rasch model (Figure 2) shows that the test provides the most information at an ability level around theta = −2, suggesting that the scale is particularly informative for individuals with lower ability levels. As theta increases, the information provided by the Rasch model gradually decreases, making it less effective for individuals with moderate to high abilities. This pattern reflects the Rasch model’s hypothesis of equal item discrimination, generally resulting in a broader but less pronounced information distribution across abilities.

### Item-fit statistics

The item-fit statistics, measured using both outfit and infit mean values, provide an overview of how well each item aligns with the expectations of the model. Items with values too high or too low may show items too easy or too difficult. The infit values ranged from 0.738 to 1.157, with a mean of 0.959 and a standard deviation of 0.132. The outfit values ranged more widely, from 0.042 to 1.096, with a mean of 0.728 and a standard deviation of 0.273. Item fit values >2 may require further review, which did not occur in this study. Both infit and outfit mean values fall within the accepted range of 0.7 to 1.3, indicating that most items fit well with the model’s expectations. These indices are not routinely applied to the 2PL model, in which discrimination parameters vary and model-based fit evaluation relies instead on global likelihood measures. Detailed infit and outfit statistics for each item are provided in Supplementary Table S2 for transparency and replicability (Table 3).

**Table 3:** Item fit statistics for the Rasch model.

Statistic	Outfit	Infit
Minimum	0.042	0.738
Maximum	1.096	1.157
Mean	0.728	0.959
Standard deviation	0.273	0.132

Summary of infit and outfit mean-square statistics across all items.

### Person-fit statistics

The person-fit statistics, calculated using outfit and infit mean values, provide insight into the consistency of individual response patterns with the expectations of the model. The infit values ranged from 0.182 to 2.290, with a mean of 0.826 and a standard deviation of 0.452. Outfit values exhibited a broader range, from 0.067 to 5.220, with a mean of 0.728 and a standard deviation of 0.809 (Table 4).

**Table 4:** Person fit statistics for the Rasch model.

Statistic	Outfit	Infit
Minimum	0.067	0.182
Maximum	5.220	2.290
Mean	0.728	0.826
Standard deviation	0.809	0.452

An overview of the infit and outfit mean-square statistics for participants.

The mean values for both infit and outfit statistics fall within the acceptable range of 0.7 to 1.3, suggesting that, on average, participants’ responses align well with model expectations.

#### 2-PL analysis

In the 2PL model, item difficulties were broadly consistent with Rasch estimates, though some items exhibited greater separation (Table 2). Discrimination values varied considerably, ranging from a high of 11.1 in item 7, indicating strong differentiation between ability levels, to a low of 0.65 in item 8, indicating limited contribution to distinguishing student performance.

**Figure 3:**
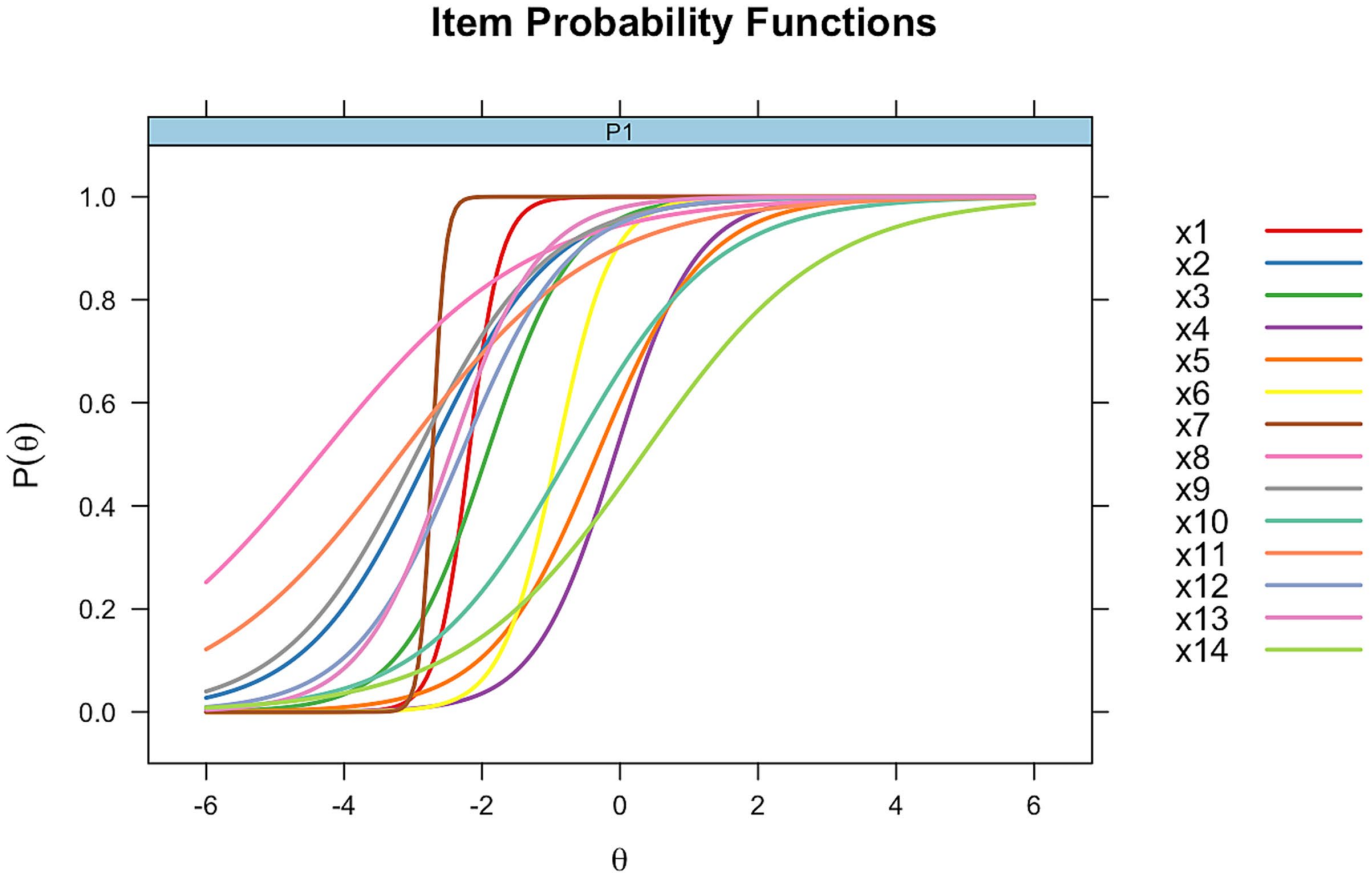
Item characteristics curves for 2PL model. Distinct slopes illustrate variable item discrimination.

Figure 3 displays the ICCs for the 2PL. In this model, items have distinct slopes that reflect their discrimination ability. For example, item 7 has a high discrimination of 11.1, indicating it is effective at distinguishing between ability levels, while item 8 has a lower discrimination of 0.65, suggesting it contributes less to differentiating individuals. Difficulty estimates also differ, with item 1 having a greater difficulty in the 2PL model (9.45) compared to the Rasch model (4.61), and item 7 showing an increase in difficulty from 6.21 in the Rasch model to 30.2 in the 2PL model. This variability in slopes and difficulty values highlights the flexibility of the 2PL model in capturing differences in item performance.

**Figure 4:**
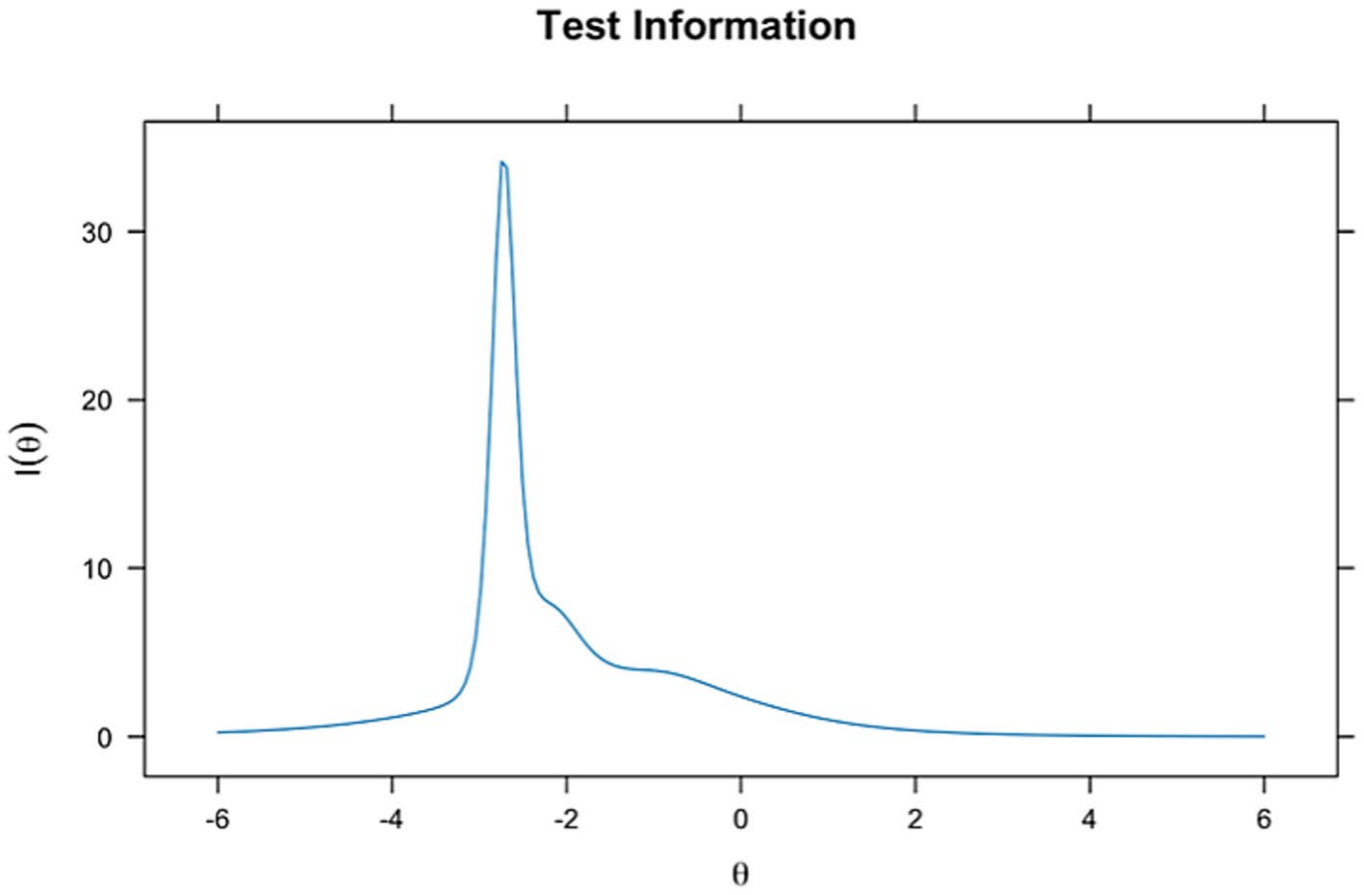
Item information curve for 2PL model.

The TIF for the 2PL model (Figure 4) also peaks around theta = −2 but exhibits a sharper peak compared to the Rasch model, suggesting that the 2PL model provides concentrated information at this specific ability level. However, the information declines outside this range, making the 2PL model less consistent across other ability levels. The sharpness of this peak is likely due to the high discrimination of certain items, such as item 7, which has a high discrimination parameter (11.1). This allows the 2PL model to be informative for lower ability levels but less effective at moderate and higher levels.

### Reliability

The reliability of the scale was evaluated using both global and ability-dependent indices. Global internal consistency was evaluated through McDonald’s *ω* and Cronbach’s *α*, computed in FACTOR software. McDonald’s ω was 0.841, and Cronbach’s α was 0.837, indicating good internal consistency and suggesting that the scale provides a reliable measurement of the underlying construct in a dichotomous item context.

**Figure 5:**
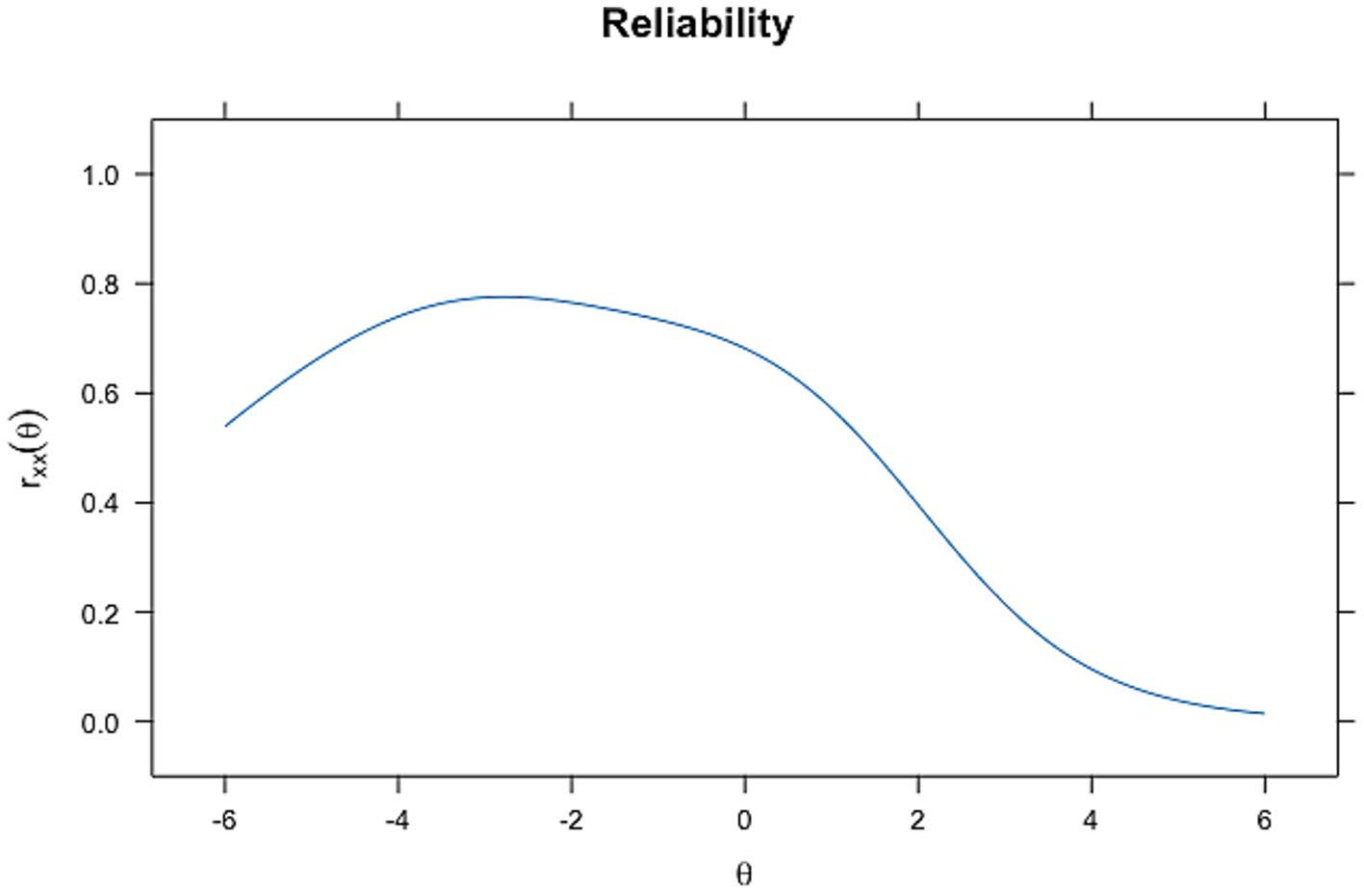
Reliability plot for Rasch analysis.

**Figure 6:**
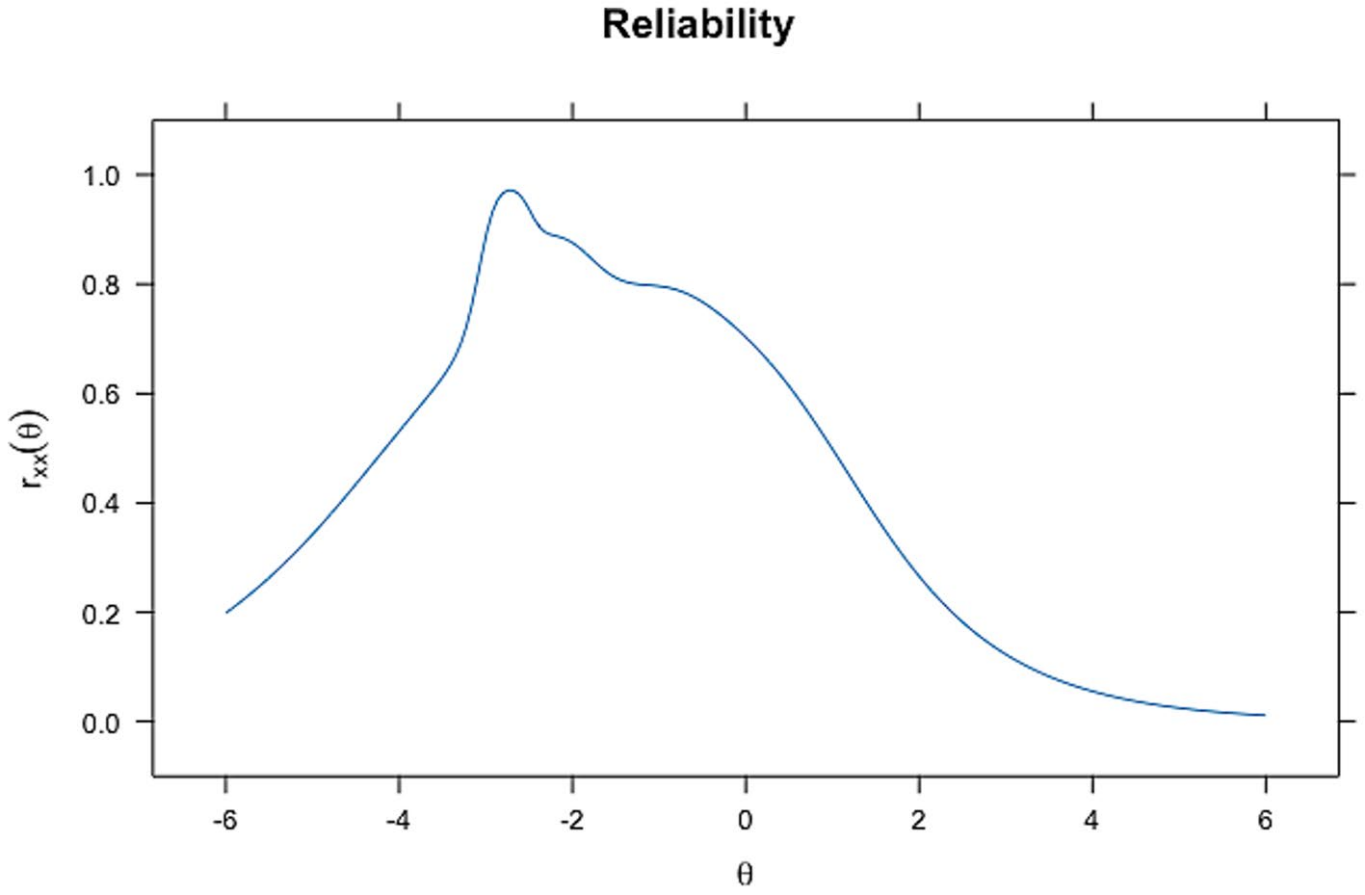
Reliability plot for 2PL analysis.

In contrast, ability-dependent reliability, derived from the *test information function* of the IRT models, varies along the latent trait (*θ*). Figures 5, 6 demonstrate this relationship. In the reliability plot for the Rasch model (Figure 5), reliability peaks at around theta = −2, reaching values close to 0.8, indicating high reliability for individuals with abilities near this level. The 2-PL model (Figure 6) also shows a peak in reliability around theta = −2, similar to the Rasch model, almost reaching a value of 1, but with a sharper increase and a slightly more varied curve shape. These findings are supported by the higher values of discrimination for Rasch and 2PL seen before.

### Item-person map

The person-item map, also known as a Wright map, provides a visual illustration of the relationship between item difficulty and students’ ability along a latent trait, in this case, IC delivery ability. In this map, the *x*-axis represents the theta, ranging from lower ability levels on the left to higher ability levels on the right. The *y*-axis displays individual items (labeled x1 to x14), each represented by a dot, with the position of the dot along the *x*-axis indicating the difficulty level of that item. Items positioned to the right are more difficult, while items on the left are easier.

At the top of the map, a histogram shows the distribution of students’ abilities. Each bar in the histogram denotes the number of students at a specific ability level. By comparing the distribution of items and participants on the same scale, the person-item map shows how well the item difficulties match the range of abilities. This alignment helps assess whether the test covers the entire ability spectrum of the sample and identifies any areas where items may be too easy or too difficult for certain respondents.

**Figure 7:**
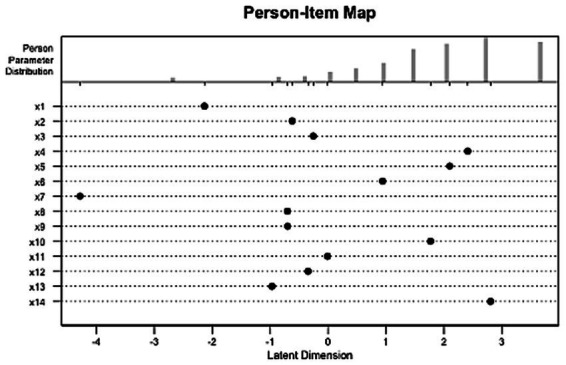
Person-item map (Wright map) for ICAS. This map displays the distribution of item difficulties (dots) and student abilities (histogram) along the latent trait (θ).

The person-item map, shown in Figure 7, reveals a good alignment between the item’s difficulty and the ability levels, with most items and person abilities concentrated around the center of the latent dimension, approximately from −1 to +2. This indicates that the test is well-suited for assessing students with moderate abilities (Table 5).

**Table 5:** Model fit comparison between the Rasch and 2PL models for the ICAS.

Model	AIC	SABIC	HQ	BIC	logLik	X^2^	df	*p*
Rasch	4498.76	512.96	4523.11	4560.56	−2234.38	NA	NA	NA
2PL	4465.31	4491.81	4510.76	4580.68	−2204.65	59.45	13	<0.0001

### Model comparison

To determine which model best represented the data, the two models were compared. First, the Akaike information criterion (AIC), the Bayesian information criterion (BIC), the sample adjusted BIC (SABIC), and the Hannan–Quinn criterion (HQ). These criteria serve to balance model fit with complexity, where lower values indicate a better fit. The AIC and BIC are useful in comparing models. Across all of these measures, lower values reflect a more optimal model that explains the data without overfitting. Additionally, the log-likelihood (logLik) was reported for each model. Log-likelihood measures the likelihood of observing the data given the model, with higher values indicating a better fit. To further quantify the difference in fit between the two models, a chi-square test (X2) was performed. A significant chi-square result indicates that one model provides a significantly better fit than the other.

Based on the results, the 2PL model showed a consistently better fit than the Rasch model, as reflected by lower AIC, BIC, SABIC, and HQ values. The chi-square test also confirmed that the 2PL model’s improvement in fit was statistically significant. This suggests that the 2PL model, which allows item discrimination to vary, captures the data’s complexity more effectively than the Rasch model, which assumes uniform discrimination across items.

### Correlation with the Minho-communication assessment scale

The results of the correlation analysis between the various assessment measures for communication skills are summarized in Table 6. The Global ICAS assessment demonstrated a strong positive correlation with the Sum of ICAS items (i.e., the total score obtained by assigning a value of 1 to each *Yes* response and 0 to each *No* response; Spearman’s *ρ* = 0.763, *p* < 0.001), suggesting that the overall ICAS score reflects the cumulative assessment of individual items well. Furthermore, Global ICAS showed an identical strong correlation with Rasch theta (ρ = 0.763, *p* < 0.001) and a similarly high correlation with 2PL theta (ρ = 0.761, *p* < 0.001). These findings indicate that both the Rasch and 2PL models effectively capture the underlying latent trait of IC skills in alignment with the global assessment.

**Table 6:** Spearman’s rho correlation matrix between the Global ICAS assessment, CAS total scores (evaluated by faculty and standardized patients), ICAS item sum, and theta values from Rasch and 2PL models.

Variable		Global	CAS total Faculty	CAS total SP	sum	Rasch	2PL
1. Global	Spearman’s rho	—					
	*p*-value	—					
2. CAS Total Faculty	Spearman’s rho	0.505	—				
	*p*-value	<0.001	—				
3. CAS Total SP	Spearman’s rho	0.244	0.243	—			
	*p*-value	<0.001	<0.001	—			
4. sum	Spearman’s rho	0.763	0.444	0.287	—		
	*p*-value	<0.001	<0.001	<0.001	—		
5. Rasch	Spearman’s rho	0.763	0.444	0.287	1.000	—	
	*p*-value	<0 0.001	<0.001	<0.001	<0.001	—	
6. 2PL	Spearman’s rho	0.761	0.452	0.282	0.970	0.970	—
	*p*-value	<0.001	<0 0.001	<0.001	<0.001	<0.001	—

The CAS Total Faculty score also showed moderate correlations with Global ICAS (ρ = 0.505, *p* < 0.001) and Sum (ρ = 0.444, *p* < 0.001), suggesting that faculty ratings align reasonably well with the ICAS-based assessments. In contrast, the CAS Total SP score exhibited a lower correlation with Global ICAS (ρ = 0.244, *p* < 0.001) and Sum (ρ = 0.287, *p* < 0.001), reflecting weaker agreement between SP evaluations and ICAS scores. These differences suggest that faculty and SPs may have different perspectives or assessment criteria in evaluating communication skills.

The Sum of ICAS items showed a perfect correlation with Rasch theta (ρ = 1.000) and a robust correlation with 2PL theta (ρ = 0.970, *p* < 0.001), underscoring the high consistency between the summed ICAS scores and the latent trait estimates generated by both models. The nearly perfect correlation between Rasch and 2PL theta values (ρ = 0.970, *p* < 0.001) further highlights the similarity in the underlying trait measurements provided by these models.

### Correlation

#### Comparison between assessed years

A total of 456 students were assessed across three academic years: Year 1 (158), Year 2 (150), and Year 3 (148). Differences across academic years were studied using the chi-square test for individual dichotomous ICAS items and one-way ANOVA for continuous outcomes (Global, Sum, Rasch, and 2PL scores).

The analysis of differences across the three academic years for individual ICAS items and overall assessment scores is presented in Tables 7, 8. Chi-squared tests identified significant differences for Items x10, x11, and x14 (*p* < 0.05). Post-hoc pairwise comparisons indicated that Year 3 differed significantly from Years 1 and 2 in Items x10 and x14, whereas Item x11 showed a significant difference between Year 2 and Year 3.

**Table 7:** Comparison of ICAS item performance between academic years (Chi-square tests).

ICAS item	χ^2^	df	*p*-value
x1	0.218	2	0.897
x2	3.840	2	0.147
x3	0.288	2	0.866
x4	5.227	2	0.073
x5	0.847	2	0.655
x6	3.654	2	0.161
x7	4.567	2	0.102
x8	1.812	2	0.404
x9	2.303	2	0.316
x10	10.202	2	0.006
x11	7.006	2	0.030
x12	1.993	2	0.369
x13	0.414	2	0.813
x14	10.006	2	0.007

**Table 8:** Comparison of continuous assessment variables between academic years.

Variable	F (2, df)	*p-*value	*Post-hoc* (Tukey)
Global ICAS	2.919 (2.455)	0.055	Year 3 > Year 1 (*p* = 0.043)
CAS Total Fac	6.237 (2.455)	0.002	Year 3 > Years 1 and 2 (*p* = 0.017, 0.005)
CAS Total SP	3.776 (2.455)	0.024	Year 3 > Year 1 (*p* = 0.019)
Sum of ICAS items	2.092 (2.455)	0.125	–
Rasch θ	2.404 (2.455)	0.092	–
2PL θ	1.015 (2.455)	0.363	–

ANOVA results comparing mean scores across three academic years for global and model-based ICAS outcomes and for external communication assessments.

For continuous variables, ANOVA and corresponding Kruskal–Wallis tests identified significant differences for the CAS Total (faculty) and CAS Total (SP) scores (*p* < 0.05). Post-hoc analysis indicated that Year 3 students achieved higher scores than Years 1 and 2 for CAS Total (faculty), and higher scores than Year 1 for CAS Total (SP).

In contrast, all other variables—including Global ICAS, sum of ICAS items, Rasch *θ*, and 2PL θ—showed no statistically significant differences across years, indicating the stability of these measures over time.

## Discussion and conclusion

### Discussion

The development and validation of the ICAS represent an important advancement in systematically assessing CS, specifically in the process of obtaining IC during medical training.

Although communication skills are widely assessed in undergraduate medical education, existing tools generally measure broad clinical communication or focus on specific contexts, such as breaking bad news. Few instruments explicitly address the unique cognitive, ethical, and interpersonal demands of IC. By centering the ICAS on observable behaviors essential to this process, such as verifying patient understanding, explaining risks and alternatives, and addressing emotions, the scale fulfills a critical gap in assessment practice.

The structured expert review conducted in the development of the ICAS ensured that each item reflected the core elements of IC communication. Unlike general communication checklists, which evaluate broad domains such as rapport-building or information-giving, the ICAS focuses on behaviors directly linked to respecting patient autonomy and promoting informed decision-making. This provides educators with a dedicated tool that complements, rather than duplicates, existing communication assessments. Moreover, the scale also supports more targeted feedback by identifying specific steps students may omit or perform inadequately during the consent process.

The internal structure analysis demonstrated that the ICAS performs as a unidimensional measure, with all indices supporting essential unidimensionality. This is consistent with the scale’s intent to measure a single underlying construct: the ability to deliver informed consent effectively. The IRT analyses revealed substantial variability in item difficulty and discrimination, particularly within the 2PL model. Such variability is educationally meaningful. For example, item 7, which involves explaining the recommended surgical treatment, exhibited extremely high discrimination, indicating that this behavior differentiates clearly between higher- and lower-performing students. Conversely, items with lower discrimination may reflect routine behaviors that most students perform consistently, contributing less to identifying differences in ability. These insights allow educators to identify which aspects of the consent process are most challenging and may require additional teaching emphasis.

The 2PL model provided a superior fit compared with the Rasch model, suggesting that allowing item discrimination to vary captures important nuances in students’ performance. This reinforces the value of modern psychometric approaches in assessing communication skills. Traditional checklist-based assessment typically assumes that items contribute equally to overall performance, but the present findings show that certain informed consent behaviors are more diagnostically informative than others. By applying IRT, the ICAS moves beyond binary scoring and provides a more precise and interpretable model of student competence.

Concurrent validity findings confirm that the ICAS relates meaningfully to broader communication assessments while measuring a more specific construct. As expected, correlations with general communication scales were moderate, reflecting conceptual overlap yet also underscoring that informed consent involves a distinct set of skills not fully captured by generic tools. Standardized patients demonstrated weaker correlations than faculty, a pattern reported in other OSCE-based studies. SP ratings often emphasize empathy and relational behaviors, whereas faculty tend to prioritize informational accuracy and structure—core elements of informed consent. This discrepancy highlights the potential value of using both perspectives when providing feedback to learners.

Cohort comparisons revealed stability across academic years in the global ICAS scores and latent trait estimates, supporting the scale’s consistency. However, specific items showed improvement in the most recent cohort, which may reflect curricular enhancements or increased student familiarity with structured consent processes. These findings offer opportunities for educators to monitor longitudinal trends and evaluate the effectiveness of teaching interventions.

Collectively, the ICAS offers distinct advantages over existing tools. The scale provides a focused, validated framework for evaluating IC, incorporates modern psychometric methods to enhance interpretability, and generates data that can support both summative decisions and formative feedback. Its specificity makes it especially useful for OSCE stations assessing ethically sensitive tasks. Moreover, by identifying items that most effectively discriminate across ability levels, the scale can guide targeted educational improvements and support curriculum design.

Despite these strengths, certain limitations should be acknowledged. The study was conducted at a single institution within a specific clinical scenario, which may limit the generalizability of the findings. Future research should evaluate the ICAS across diverse clinical contexts, across institutions, and among postgraduate learners. In addition, examining predictive validity, such as the relation between ICAS performance and real-world consent interactions, would further strengthen its utility.

Overall, the ICAS represents a meaningful contribution to medical education assessment by offering a reliable, valid, and context-sensitive tool for evaluating IC communication skills. Its integration into OSCEs can enhance both assessment accuracy and the quality of feedback provided to trainees, ultimately supporting more competent and ethically grounded clinical practice.

### Conclusion and practice implications

The ICAS addresses the need for a customized tool to evaluate obtaining IC, as it measures the competencies that are considered essential for proficiency in this clinical act.

The application of IRT was instrumental in the scale’s development, providing detailed insights into item performance and enabling precise measurement of student ability. Analyses conducted with both the Rasch and 2PL models confirmed the scale’s effectiveness, with the 2PL model demonstrating superior fit by capturing variability in item discrimination. Evidence of moderate reliability and strong validity supports the ICAS as a stable, scenario-specific assessment tool.

Beyond assessment, the ICAS serves as a valuable instrument for delivering targeted feedback, enabling students to refine their communication approaches in high-stakes clinical interactions. Future studies should explore the use of the ICAS across diverse educational settings to maximize its impact and utility.

## Data Availability

The original contributions presented in the study are included in the article/Supplementary material, further inquiries can be directed to the corresponding author.

## References

[1] Wancata LM Morgan H Sandhu G Santen S Hughes DT. Using the ACMGE milestones as a handover tool from medical school to surgery residency. J Surg Educ. (2017) 74:519–29. doi: 10.1016/j.jsurg.2016.10.016, 27908638

[2] Frank J. A history of CanMEDS - chapter from Royal College of Physicians of Canada 75th anniversary history. Ottawa (2004).

[3] Moral RR Andrade-Rosa C Molina JD Barba EC de Torres LP Martín DM. Effectiveness of a communication skills training program for medical students to identify patients communicative clues. Patient Educ Couns. (2020) 103:2384–7. doi: 10.1016/j.pec.2020.05.018, 32451220

[4] Nakagawa S Fischkoff K Berlin A Arnell TD Blinderman CD. Communication skills training for general surgery residents. J Surg Educ. (2019) 76:1223–30. doi: 10.1016/j.jsurg.2019.04.001, 31005480

[5] Taylor LJ Johnson SK Nabozny MJ Tucholka JL Steffens NM Kwekkeboom KL . Barriers to goal-concordant Care for Older Patients with Acute Surgical Illness: communication patterns extrinsic to decision aids. Ann Surg. (2018) 267:677–82. doi: 10.1097/SLA.0000000000002282, 28448386 PMC6544363

[6] Chandawarkar RY Ruscher KA Krajewski A Garg M Pfeiffer C Singh R . Pretraining and Posttraining assessment of residents’ performance in the fourth accreditation Council for Graduate Medical Education Competency: patient communication skills. Arch Surg. (2011) 146:916–21. doi: 10.1001/archsurg.2011.167, 21844435

[7] Zerbini G Reicherts P Reicherts M Roob N Schneider P Dankert A . Communication skills of medical students: evaluation of a new communication curriculum at the University of Augsburg. GMS J Med Educ. (2024) 41:Doc26. doi: 10.3205/zma001681 39131896 PMC11310792

[8] Ha JF Longnecker N. Doctor-patient communication: a review. Ochsner J. (2010) 10:38. 21603354 PMC3096184

[9] Anderson TN Kaba A Gros E Schmiederer IS Shi R Aalami LR . A novel blended curriculum for communication of informed consent with surgical interns. J Grad Med Educ. (2021) 13:411–6. doi: 10.4300/JGME-D-20-01057.1, 34178267 PMC8207932

[10] Zolnierek KBH DiMatteo MR. Physician communication and patient adherence to treatment: a meta-analysis. Med Care. (2009) 47:826. doi: 10.1097/MLR.0b013e31819a5acc, 19584762 PMC2728700

[11] Raper SE Gupta M Okusanya O Morris JB. Improving communication skills: a course for Academic Medical Center surgery residents and faculty. J Surg Educ. (2015) 72:e202–11. doi: 10.1016/j.jsurg.2015.06.008, 26183787

[12] Cömert M Zill JM Christalle E Dirmaier J Härter M Scholl I. Assessing communication skills of medical students in objective structured clinical examinations (OSCE) - a systematic review of rating scales. PLoS One. (2016) 11:e0152717. doi: 10.1371/journal.pone.0152717, 27031506 PMC4816391

[13] Trickey AW Newcomb AB Porrey M Wright J Bayless J Piscitani F . Assessment of surgery residents’ interpersonal communication skills: validation evidence for the communication assessment tool in a simulation environment. J Surg Educ. (2016) 73:e19–27. doi: 10.1016/j.jsurg.2016.04.016, 27216300

[14] Horwitz IB Horwitz SK Brandt ML Brunicardi FC Scott BG Awad SS. Assessment of communication skills of surgical residents using the social skills inventory. Am J Surg. (2007) 194:401–5. doi: 10.1016/j.amjsurg.2006.11.039, 17693291

[15] Wood F Martin SM Carson-Stevens A Elwyn G Precious E Kinnersley P. Doctors’ perspectives of informed consent for non-emergency surgical procedures: a qualitative interview study. Health Expectations: Int J Public Participation Health Care Health Policy. (2014) 19:751–61. doi: 10.1111/hex.12258, 25212709 PMC5055244

[16] Raper SE Joseph J. Informed consent for academic surgeons: a curriculum-based update. MedEdPORTAL: J Teach Learn Resources. (2020) 16:10985. doi: 10.15766/mep_2374-8265.10985, 33015359 PMC7528671

[17] Chia CLK Chan KS Ng MJM Rao AD Singaporewalla R. Assessing adequacy of informed consent for elective surgery by student-administered interview. ANZ J Surg. (2019) 89:677–82. doi: 10.1111/ans.15214, 31090182

[18] Leeper-Majors K Veale JR Westbrook TS Reed K. The effect of standardized patient feedback in teaching surgical residents informed consent: results of a pilot study. Curr Surg. (2003) 60:615–22. doi: 10.1016/S0149-7944(03)00157-0, 14972204

[19] Downing MT Way DP Caniano DA. Results of a national survey on ethics education in general surgery residency programs. Am J Surg. (1997) 174:364–8. 9324157 10.1016/s0002-9610(97)00112-8

[20] Sherman KA Kilby CJ Pehlivan M Smith B. Adequacy of measures of informed consent in medical practice: a systematic review. PLoS One. (2021) 16:e0251485. doi: 10.1371/journal.pone.0251485, 34043651 PMC8159027

[21] Koller SE Moore RF Goldberg MB Zhang J Yu D Conklin CB . An informed consent program enhances surgery resident education. J Surg Educ. (2017) 74:906–13. doi: 10.1016/j.jsurg.2017.02.002, 28238705

[22] Zhang Y Jiang G Sun Y Zhao X Yu X. Adaptation of the communication skills attitude scale (CSAS) to surgical residents in China. J Surg Educ. (2019) 76:329–36. doi: 10.1016/j.jsurg.2018.07.027, 30139694

[23] Stucke R Rosenkranz KM. Teaching and evaluating nontechnical skills for general surgery. Surg Clin N Am. (2021) 101:577–86. doi: 10.1016/j.suc.2021.05.005, 34242601

[24] Brasel KJ Klingensmith ME Englander R Grambau M Buyske J Sarosi G . Entrustable professional activities in general surgery: development and implementation. J Surg Educ. (2019) 76:1174–86. doi: 10.1016/j.jsurg.2019.04.003, 31029575

[25] White EM Miller SM Esposito AC Yoo PS. “Let’s get the consent together”: rethinking how surgeons become competent to discuss informed consent. J Surg Educ. (2020) 77:e47–51. doi: 10.1016/j.jsurg.2020.07.028, 32753261

[26] Turner JL Dankoski ME. Objective structured clinical exams: a critical review. Fam Med. (2008) 40:574–8. 18988044

[27] Talwalkar JS Auguste H Fortin VI Morrison LJ Kliger A Rosenthal DI Murtha T . An advanced communication skills workshop using standardized patients for senior medical students. MedEdPORTAL: J Teach Learn Resources. (2021) 17:11163. doi: 10.15766/mep_2374-8265.11163 PMC8155077 34124349

[28] Piumatti G Cerutti B Perron NJ. Assessing communication skills during OSCE: need for integrated psychometric approaches. BMC Med Educ. (2021) 21:106. doi: 10.1186/s12909-021-02552-8, 33593345 PMC7887794

[29] Wilby KJ Govaerts MJB Dolmans DHJM Austin Z van der Vleuten C. Reliability of narrative assessment data on communication skills in a summative OSCE. Patient Educ Couns. (2019) 102:1164–9. doi: 10.1016/j.pec.2019.01.018, 30711383

[30] Kurtz SM Silverman JD. The Calgary-Cambridge referenced observation guides: an aid to defining the curriculum and organizing the teaching in communication training programmes. Med Educ. (1996) 30:83–9. 8736242 10.1111/j.1365-2923.1996.tb00724.x

[31] Makoul G Krupat E Chang C-H. Measuring patient views of physician communication skills: development and testing of the communication assessment tool. Patient Educ Couns. (2007) 67:333–42. doi: 10.1016/j.pec.2007.05.005, 17574367

[32] Baile WF Buckman R Lenzi R Glober G Beale EA Kudelka AP. SPIKES-A six-step protocol for delivering bad news: application to the patient with cancer. Oncologist. (2000) 5:302–11. doi: 10.1634/theoncologist.5-4-302, 10964998

[33] Gonçalves M Gonçalves N Mendonça-Gonçalves M Sousa AL Morgado P Sousa N . Minho communication assessment scale: development and validation. Acta Medica Port. (2020) 33:326–34. doi: 10.20344/amp.12727, 32416755

[34] De Champlain AF. A primer on classical test theory and item response theory for assessments in medical education. Med Educ. (2010) 44:109–17. doi: 10.1111/j.1365-2923.2009.03425.x, 20078762

[35] Downing SM. Item response theory: applications of modern test theory in medical education. Med Educ. (2003) 37:739–45. doi: 10.1046/j.1365-2923.2003.01587.x, 12945568

[36] Morgado FFR Meireles JFF Neves CM Amaral ACS Ferreira MEC. Scale development: ten main limitations and recommendations to improve future research practices. Psicol Reflex Crit. (2017) 30:3. doi: 10.1186/s41155-016-0057-1, 32025957 PMC6966966

[37] Boateng GO Neilands TB Frongillo EA Melgar-Quiñonez HR Young SL. Best practices for developing and validating scales for health, social, and behavioral research: a primer. Front Public Health. (2018) 6:149. doi: 10.3389/fpubh.2018.00149, 29942800 PMC6004510

[38] Ilgen JS Ma IWY Hatala R Cook DA. A systematic review of validity evidence for checklists versus global rating scales in simulation-based assessment. Med Educ. (2015) 49:161–73. doi: 10.1111/medu.12621, 25626747

[39] Ferrando Pere J. Lorenzo-Seva Urbano, (2018). Assessing the quality and appropriateness of factor solutions and factor score estimates in exploratory item factor analysis. Available online at: https://journals.sagepub.com/doi/10.1177/0013164417719308 (accessed April 16, 2025). 10.1177/0013164417719308 PMC7328234 32655169

[40] Ferrando P Lorenzo-Seva U. Program FACTOR at 10: origins, development and future directions. Psicothema. (2017) 2:236–40. doi: 10.7334/psicothema2016.304, 28438248

