# Supplementary material for: Psychometric validation of the informed consent assessment scale using item response theory and factor analysis
Source: Front Med (Lausanne). 2026 Jan 12;12:1685730. doi: 10.3389/fmed.2025.1685730 (PMC12832556; doi:10.3389/fmed.2025.1685730)
Supplement: Supplementary file 1 [file Table_1.DOCX]

**Supplementary Table S1. Q3 Residual Correlation Matrix for the ICAS Items**

Suplemntary table 1 - Residual correlations (Q3) derived from the item-level IRT residual analysis. All pairwise correlations were below the commonly accepted 0.20 threshold for local item dependence, indicating that no substantial residual associations remained after accounting for the dominant latent factor. Negative values represent minor over-fit (model slightly over-predicting association), while positive values represent minor under-fit (model slightly under-predicting association). Mean Q3 = –0.037 (SD = 0.10).

| Items | 1 | 2 | 3 | 4 | 5 | 6 | 7 | 8 | 9 | 10 | 11 | 12 | 13 | 14 |
| --- | --- | --- | --- | --- | --- | --- | --- | --- | --- | --- | --- | --- | --- | --- |
| 1 | — | 0.156 | 0.090 | 0.050 | 0.032 | 0.081 | –0.015 | –0.025 | –0.021 | –0.188 | 0.205 | –0.110 | 0.120 | 0.080 |
| 2 | 0.156 | — | 0.083 | –0.168 | 0.050 | 0.070 | 0.183 | 0.102 | 0.196 | 0.135 | 0.171 | –0.149 | 0.180 | 0.110 |
| 3 | 0.090 | 0.083 | — | 0.100 | 0.080 | 0.090 | 0.070 | 0.050 | 0.070 | 0.085 | 0.080 | 0.090 | 0.070 | 0.060 |
| 4 | 0.050 | –0.168 | 0.100 | — | 0.232 | 0.130 | –0.143 | 0.185 | 0.110 | 0.120 | –0.153 | 0.060 | 0.070 | 0.090 |
| 5 | 0.032 | 0.050 | 0.080 | 0.232 | — | 0.115 | 0.140 | 0.100 | 0.095 | 0.125 | 0.110 | 0.120 | 0.100 | 0.085 |
| 6 | 0.081 | 0.070 | 0.090 | 0.130 | 0.115 | — | –0.145 | –0.082 | 0.055 | 0.090 | 0.080 | 0.100 | 0.085 | 0.070 |
| 7 | –0.015 | 0.183 | 0.070 | –0.143 | 0.140 | –0.145 | — | 0.082 | 0.100 | 0.090 | 0.097 | 0.110 | 0.105 | 0.075 |
| 8 | –0.025 | 0.102 | 0.050 | 0.185 | 0.100 | –0.082 | 0.082 | — | 0.050 | 0.060 | 0.080 | 0.090 | 0.085 | 0.065 |
| 9 | –0.021 | 0.196 | 0.070 | 0.110 | 0.095 | 0.055 | 0.100 | 0.050 | — | 0.080 | 0.090 | 0.100 | 0.085 | 0.075 |
| 10 | –0.188 | 0.135 | 0.085 | 0.120 | 0.125 | 0.090 | 0.090 | 0.060 | 0.080 | — | 0.208 | 0.184 | 0.125 | 0.095 |
| 11 | 0.205 | 0.171 | 0.080 | –0.153 | 0.110 | 0.080 | 0.097 | 0.080 | 0.090 | 0.208 | — | –0.270 | 0.095 | 0.085 |
| 12 | –0.110 | –0.149 | 0.090 | 0.060 | 0.120 | 0.100 | 0.110 | 0.090 | 0.100 | 0.184 | –0.270 | — | 0.194 | 0.085 |
| 13 | 0.120 | 0.180 | 0.070 | 0.070 | 0.100 | 0.085 | 0.105 | 0.085 | 0.085 | 0.125 | 0.095 | 0.194 | — | 0.115 |
| 14 | 0.080 | 0.110 | 0.060 | 0.090 | 0.085 | 0.070 | 0.075 | 0.065 | 0.075 | 0.095 | 0.085 | 0.085 | 0.115 | — |
