# Supplementary material for: Psychometric validation of the informed consent assessment scale using item response theory and factor analysis
Source: Front Med (Lausanne). 2026 Jan 12;12:1685730. doi: 10.3389/fmed.2025.1685730 (PMC12832556; doi:10.3389/fmed.2025.1685730)
Supplement: Supplementary file 2 [file Table_2.DOCX]

**Supplementary Table S2. Infit and Outfit Mean-Square Statistics for Each ICAS Item (Rasch Model)**

Supplementary Table 2 - Item Fit Statistics for Rasch Model

| **Item** | **Outfit** | **z-Outfit** | **Infit** | **z-Infit** |
| --- | --- | --- | --- | --- |
| 1 | 0.258 | −1.95 | 0.996 | 0.10 |
| 2 | 0.827 | −0.58 | 1.062 | 0.43 |
| 3 | 0.740 | −1.20 | 0.981 | −0.10 |
| 4 | 0.723 | −5.63 | 0.785 | −6.02 |
| 5 | 0.774 | −4.24 | 0.847 | −3.94 |
| 6 | 0.637 | −3.69 | 0.827 | −2.65 |
| 7 | 0.042 | −3.20 | 0.738 | −0.32 |
| 8 | 1.096 | 0.42 | 1.157 | 0.93 |
| 9 | 0.834 | −0.52 | 1.088 | 0.56 |
| 10 | 0.803 | −3.11 | 0.867 | −3.02 |
| 11 | 1.001 | 0.07 | 1.101 | 0.87 |
| 12 | 0.857 | −0.56 | 1.047 | 0.38 |
| 13 | 0.740 | −0.77 | 1.047 | 0.30 |
| 14 | 0.859 | −2.48 | 0.883 | −3.14 |
